# Beneficial impact of cathelicidin on hypersensitivity pneumonitis treatment—*In vivo* studies

**DOI:** 10.1371/journal.pone.0251237

**Published:** 2021-05-17

**Authors:** Marta Kinga Lemieszek, Katarzyna Sawa-Wejksza, Marcin Golec, Jacek Dutkiewicz, Jacek Zwoliński, Janusz Milanowski

**Affiliations:** 1 Department of Medical Biology, Institute of Rural Health, Lublin, Poland; 2 Department of Virology and Immunology, Maria Curie-Skłodowska University, Lublin, Poland; 3 Unit of Fibroproliferative Diseases, Institute of Rural Health, Lublin, Poland; 4 Department of Biological Health Hazards and Parasitology, Institute of Rural Health, Lublin, Poland; 5 Department of Pneumonology, Oncology and Allergology, Medical University of Lublin, Lublin, Poland; Helmholtz-Zentrum Munich, GERMANY

## Abstract

Cathelicidin (CRAMP) is a defence peptide with a wide range of biological responses including antimicrobial, immunomodulatory and wound healing. Due to its original properties the usefulness of CRAMP in the treatment of pulmonary fibrosis was assessed in a murine model of hypersensitivity pneumonitis (HP). The studies were conducted on mouse strain C57BL/6J exposed to a saline extract of *Pantoea agglomerans* cells (HP inducer). Cathelicidin was administered in the form of an aerosol during and after HP development. Changes in the composition of immune cell populations (NK cells, macrophages, lymphocytes: Tc, Th, Treg, B), were monitored in lung tissue by flow cytometry. Extracellular matrix deposition (collagens, hydroxyproline), the concentration of cytokines involved in inflammatory and the fibrosis process (IFNγ, TNFα, TGFβ1, IL1β, IL4, IL5, IL10, IL12α, IL13) were examined in lung homogenates by the ELISA method. Alterations in lung tissue morphology were examined in mouse lung sections stained with haematoxylin and eosin as well as Masson trichrome dyes. The performed studies revealed that cathelicidin did not cause any negative changes in lung morphology/structure, immune cell composition or cytokines production. At the same time, CRAMP attenuated the immune reaction induced by mice chronic exposure to *P*. *agglomerans* and inhibited hydroxyproline and collagen deposition in the lung tissue of mice treated with bacteria extract. The beneficial effect of CRAMP on HP treatment was associated with restoring the balance in quantity of immune cells, cytokines production and synthesis of extracellular matrix components. The presented study suggests the usefulness of cathelicidin in preventing lung fibrosis; however, cathelicidin was not able to reverse pathological changes completely.

## Introduction

Hypersensitivity pneumonitis (HP), or extrinsic allergic alveolitis, is an interstitial lung disease induced by chronic exposure to organic dust (particles of plants, animals, fungi, or bacteria) [1]. HP is a complex clinical condition involving a cascade of immune reactions triggered and sustained by lung injury due to repeated inhalations of fine organic particles inhaled into the airways. The clinical course of HP is most often divided into an acute form, dominated by inflammation with macrophage-lymphocyte responses, and a chronic phase, characterized by ongoing immune activation and inflammation leading to the expansion as well as activation of the fibroblast and the accumulation of extracellular matrix responsible for fibrotic lesions in lung tissue [1]. HP comprises a number of variants, e.g. farmer’s lung, malt fever and pigeon fancier’s lung, which show a similar clinical course but are caused by different types organic dust containing a variety of specific etiological factors, including Gram-negative bacteria, fungi, thermophilic actinomycetes or bird proteins [1–6]. The pathogenic agents are mostly glycoproteins produced by dust-borne fungi and bacteria, less often animal and plant proteins [7, 8]. As mentioned, HP can be provoked by a diverse array of antigens. One of the HP causative agents worth noting is Gram-negative bacteria *Pantoea agglomerans*, which is a fermentative, epiphytic bacterium widely distributed in nature, especially on the surface of plants. It constitutes a prevailing component of the microflora of grain, cotton, herbs, flax and many other plant materials used in industry [9, 10]. *P*. *agglomerans* also predominates in inhalable dust from grain, herbs, and flax, and was identified as a premier cause of allergy in grain workers, herb workers and flax farmers [9, 10]. It has been documented that this bacterium is the most important cause of HP in eastern Poland [3, 6, 11–13]. The above-mentioned state of knowledge enabled us to create and to validate the mouse HP model [14, 15], which was used in the presented study.

Based on HP etiology, as well as mechanisms occurring during disease development, HP may be simply described as a pathology in which loss of lung function occurs due to fibrotic reaction in a highly inflammatory condition induced by chronic exposure to organic dust. Because of the fact that the main mechanisms of HP development are the pathology of repair of the damaged pulmonary epithelium, as well as chronic inflammation, it is assumed that effective therapeutic options should combine immune balance restoration with wound healing enhancement. These criteria involve encountering some host defence peptides, such as cathelicidins.

Cathelicidins (LL-37 in human and CRAMP in mice) belong to the large, conserved group of antimicrobial peptides and represent an important part of the innate immunity [16]. These host defence peptides directly kill both Gram-negative and Gram-positive bacteria as well as some enveloped viruses, parasites and fungi by perturbing their cell membranes [17, 18]. Furthermore, they can also neutralize the biological activities of endotoxin [19, 20]. Next to direct killing of pathogens, cathelicidins increase the natural abilities of immune cells to fight infection in several different ways including attraction and recognition of pathogens, enhancement of phagocytosis, stimulation of production and release of proinflammatory compounds [17, 21–25]. In addition to improving the host’s response to pathogens cathelicidins also accelerate epithelial cells proliferation, migration, and promotion of wound closure, which together play an important role in the maintenance of tissue homeostasis by supervision of regenerative processes [26–28].

To present the pleiotropic activity of cathelicidins was not the only reason for conducting the presented study. Changes in cathelicidin concentration in the course of respiratory pathologies and due to exposure to organic dust had also been previously demonstrated by the authors. The previously obtained results indicated a decrease in cathelicidin levels in the human pulmonary compartment in cases of advanced lung tissue remodelling: in pulmonary fibrosis [29, 30] and in advanced stages of COPD (Chronic Obstructive Pulmonary Disease) [31, 32]. Furthermore, a significant decrease in cathelicidin was observed in both CRAMP gene expression and cathelicidin concentration during HP development in the above-mentioned murine model of this disease [33]. Due to its original properties, cathelicidin has become a target of some therapeutic approaches; however, according to the best of the authors’ knowledge, the presented research is the first to attempt determination of the possibility of using cathelicidin in the prevention and/or treatment of pulmonary fibrosis in the course of HP, as well as the first approach in administering cathelicidin in the form of inhalations. In order to achieve the mentioned goal, the influence of inhaled cathelicidin on lung tissue in normal and pathological conditions, as well as the mechanism of it action, was investigated in the murine model of HP.

## Materials and methods

### Reagents

Unless otherwise indicated, the chemicals used in the study were purchased from Sigma-Aldrich Co. LLC.

### Cathelicidin

Murine cathelicidin (ISRLAGLLRKGGEKIGEKLKKIGQKIKNFFQKLVPQPE); purity 95.53%, was purchased from the Novozym Polska s.c. Poznań Science and Technology Park. Lyophilized peptides were resuspended in sterile distilled water with 1% BSA to a final concentration of 1μg/ml. Dissolved portions of cathelicidin (10 μl/tube) were stored at −80°C prior to using. Working solutions of CRAMP were prepared by dissolving stock solution in phosphate buffered saline (PBS) just before use. All stages of CRAMP solution preparation were carried out under sterile conditions.

### Saline extract of *Pantoea agglomerans*

*Pantoea agglomerans* strain M-10-3 was first isolated from the air of a grain mill over 30 years ago, and has been used ever since as the standard strain in the research conducted by the employees of the Institute of Rural Health in Lublin, Poland [34, 35]. In this study, the M-10-3 strain was inoculated on enriched nutrient agar medium (BTL, Łódź, Poland) supplemented with peptides (Proteobak, BTL, Łódź, Poland). The cultures were incubated in Roux bottles for 72 h at 37°C. The bacterial mass was harvested by washing off with sterile distilled water, homogenized with a glass homogenizer and extracted in saline (0.85% NaCl) in the proportion 1:2 for 48 h at 4°C, with intermittent disruption of cells by 10-fold freezing and thawing. Afterwards, the supernatant was separated by centrifugation at 10,000 rpm at 4°C, and finally lyophilized. The studies were performed using saline extract of the *P*. *agglomerans* (SE-PA), obtained by dissolving *P*. *agglomerans* cells lyophilizate in PBS just before use. All stages of SE-PA preparation were carried out under sterile conditions. SE-PA contains proteins, sugars, DNA and RNA (42.3%, 15.2%, 0.018%, and 0.014%, respectively, as determined by spectrophotometric analysis) and a relatively small quantity of active endotoxin (1% as assessed by *Limulus* test Cat. No 25003A, 32500A, 500100KE; Pyroquant Diagnostik GmbH, Germany) [9, 36].

### Animal inhalation

Three-month-old female C57BL/6J mice were purchased from Mossakowski Medical Research Centre of the Polish Academy of Sciences in Warsaw, Poland. The mice were kept in colony cages with free access to food and tap water *ad libitum*, under standardized housing conditions (natural light-dark cycle, a temperature of 22–24°C). After 7 days of adaptation to laboratory conditions, adaptation to inhalation procedures was started in inhalation chambers, which included taming with silicon masks placed on their mouths, and a collar placed on the neck of each animal which immobilized them while allowing them to breathe freely and protect against overheating. The training took place daily for 7 consecutive days. The mice were weighed daily and their health and well-being was continuously monitored by a veterinarian.

In the first step, C57BL/6J mice were exposed to a finely dispersed aerosol of phosphate buffered saline (PBS; 5 ml/ single inhalation), and then to cathelicidin at concentrations 4, 6 or 8 times higher than physiological (2.64 μg/ml; 3.96 μg/ml; 5.28 μg/ml) (Table 1). The mice were treated for one hour for each investigated factor: PBS and CRAMP. Inhalations were conducted using the Buxco Inhalation Tower (Data Sciences International, St. Paul, USA) under the following conditions: air flow 1.5 l/min; pressure 0.5 cm H_2_O; room temperature; nebulization rate 84 μl/min. Both untreated (control) and treated mice were sacrificed by cervical dislocation with spinal cord rupture; lung samples were then collected in order to determine the physiological concentration of CRAMP (untreated mice), as well as amount of CRAMP, in response to a single inhalation with exogenous cathelicidin at the above-mentioned concentrations.

**Table 1:** Description of research groups.

Name of research group (No. of animals)	PBS (exposure time)	SE-PA (exposure time)	CRAMP (exposure time)	Factors administration sequence
**Untreated (n = 20)**	-	-	-	-
**4xCRAMP (n = 4)**	1 h	-	1 h exposure to CRAMP at 4x physiologic concentration	same day, consecutively
**6xCRAMP (n = 4)**	1 h	-	1 h exposure to CRAMP at 6x physiologic concentration	same day, consecutively
**8xCRAMP (n = 4)**	1 h	-	1 h exposure to CRAMP at 8x physiologic concentration	same day, consecutively
**CRAMP 14 d. (n = 6)**	1 h/day during 14 days	-	1 h/day during 14 days	same day; consecutively
**CRAMP 28 d. (n = 6)**	1 h/day during 28 days	-	1 h/day during 28 days	same day; consecutively
**SE-PA 14 d. (n = 6)**	1 h/day during 14 days	1 h/day during 14 days	-	same; day, consecutively
**SE-PA 28 d. (n = 6)**	1 h/day during 28 days	1 h/day during 28 days	-	same day; consecutively
**SE-PA+CRAMP 14 d. (n = 6)**	-	1 h/day during 14 days	1 h/day during 14 days	same day; consecutively
**SE-PA+CRAMP 28 d. (n = 6)**	-	1 h/day during 28 days	1 h/day during 28 days	same day; consecutively
**SE-PA 28 d. + untreated 14 d. (n = 6)**	1 h/day during 28 days	1 h/day during 28 days	-	same day; consecutively
	-	-	-	after 28 days of treatment mice stay an additional 14 days in the experiment without exposure
**SE-PA 28 d. + CRAMP 14 d. (n = 6)**	1 h/day during 28 days	1 h/day during 28 days	-	same day; consecutively
			1 h/day; during 14 days	after 28 days of mice exposure to (PBS + SE-PA), CRAMP was applied for an additional 14 days

PBS—phosphate buffered saline; SE-PA—saline extract of *Pantoea agglomerans*; CRAMP–cathelicidin; d.–days; n–number of animals in research group.

C57BL/6J mice were exposed to a finely dispersed aerosol of the saline extract of *P*. *agglomerans* (SE-PA; 10 mg/ml; 5 ml/ single inhalation), cathelicidin (CRAMP; 1.44 μg/ml; 5 ml/ single inhalation) or phosphate buffered saline (PBS; 5 ml/ single inhalation), administered separately or in combinations (Table 1). The mice were treated to each investigated factor for one hour daily for 14, 28 or 42 days. Inhalations were carried out using the Buxco Inhalation Tower under the above-mentioned conditions. Both untreated (control) and treated mice were sacrificed by cervical dislocation with spinal cord rupture, and lung samples collected. Lung samples for histological examination was fixed in 4% buffered formalin, lung samples for protein analysis were frozen at -20°C until evaluation, while lung samples for cell counting were analyzed immediately after collection.

Before treatment and after every 7 days of exposure to the investigated factors, the animals were placed in a Non-invasive Airway Mechanics Plethysmograph Chamber with Allay Restraint and Halcon Technology (Data Sciences International, St. Paul, USA). Pulmonary function of the restrained mice were monitored under the following conditions: air flow 0.6 l/min; pressure 0 cm H_2_O; at room temperature. The data were collected every 2 s for 15 min. The frequency of breathing (f) and tidal volume (TV) were determined using DSI FinePointe Software.

The experimental protocols were approved by the Local Ethics Committee for Animal Experimentation in Lublin, Poland (Resolution Nos. 39/2016 and 2/2017). Due to the mild severity of the procedures performed, the animals did not receive sedatives or analgesics.

Design of the experiments is presented in S1 Fig.

### Examination for signs of inflammation and fibrosis in lung tissue—haematoxylin and eosin staining and Masson trichrome staining

Lung tissue was fixed in 4% buffered formalin for 12 h, followed by dehydration in an ascending series of alcohol and embedding in paraffin wax. 5 μm thick sections were obtained from the paraffin blocks and stained with haematoxylin and eosin (H&E) or Masson trichrome. Micrographs were evaluated by light microscopy to assess their general morphology (H&E staining) and to detect collagen fibres (Masson trichrome staining). Histological examination was performed by a pathologist who was blinded to the experimental protocol. Lung injury was scored according to features of inflammation and fibrosis. These features were graded with 4 point scales: 0 = regular tissue, 1 = mild changes, 2 = moderate changes, 3 = significant changes. Mentioned parameters were analyzed twice and the final scores were calculated as the mean of investigated items in each research group. Lung tissue images were captured with microscopy Olympus BX51 System Microscope at magnification 200x. Obtained images were analysed using AnalySIS software.

### Measurement of selected proteins concentration–ELISA method

Lung samples were placed in Lysing Matrix M tubes (Cat. No 116923500, MP Biomedicals, Solon, USA). Cell Disruption Buffer (Cat. No AM1921, PARIS Kit; Life Technologies, Carlsbad, USA) was added and tissues homogenized mechanically using a FastPrep-24 5G homogeniser (MP Biomedicals) under the following conditions: 6 m/s, 40 s, 20°C. The homogenates were further incubated on ice for 20 min. The homogenates were next passed through a 70 μm nylon mesh and centrifugated (10,000 x G, 5 min., 4°C). Supernatants were collected in new tubes and their protein content was determined by standard BCA Protein Assay Kit (Cat. No 23227, Pierce Biotechnology, Rockford, USA). Collected homogenates were stored at -80°C for later use. The concentration of selected proteins in lung tissue homogenates containing 25 μg of total proteins were measured by the following ELISA kits: Mouse Hydroxyproline (Hyp) Cat. No CEA621Ge; Mouse Collagen Type I (COL1) Cat. No SEA571Mu; Mouse Collagen Type III (COL3) Cat. No SEA176Mu; Mouse Collagen Type IV (COL4) Cat. No SEA180Mu; Mouse Cathelicidin Antimicrobial Peptide (CAMP) Cat. No SEC419Mu; Mouse Interleukin 1 Beta (IL1b) Cat. No SEA563Mu; Mouse Interleukin 4 (IL4) Cat. No SEA077Mu; Mouse Interleukin 5 (IL5) Cat. No SEA078Mu; Mouse Interleukin 10 (IL10) Cat. No SEA056Mu; Mouse Interleukin 12A (IL12A) Cat. No SEA059Mu; Mouse Interleukin 13 (IL13) Cat. No SEA060Mu; Mouse Interferon Gamma (IFNg) Cat. No SEA049Mu; Mouse Tumour Necrosis Factor Alpha (TNFa) Cat. No SEA133Mu; Mouse Transforming Growth Factor Beta 1 (TGFb1) Cat. No SEA124Mu (Cloud-Clone Corp., USA), according to manufacturer’s instructions.

### Investigation of selected immune cell subpopulations in lung tissue homogenates—flow cytometry

Lung samples were placed in a cold RPMI cell culture medium supplemented with 10% FBS (fetal bovine serum) and finely minced into using a surgical blade. The tissue was then incubated in RPMI with collagenase type IV Cat. No C5138 (0.5 mg/ml), DNAse I Cat. No 11284932001 (25 u/ml), and elastase Cat. No E7885 (25 μg/ml) for 45 min in 20˚C. Obtained lung tissue homogenates were passed through a 70-μm filter and centrifugated (500 x G; 10 min. 20˚C). Erythrocytes present in the obtained cell mixture were removed using Human Erythrocyte Lysing Kit (Cat. No WL1000, R&D Systems, Inc. UK). Next, the obtained cells were suspended in RPMI supplemented with 10% FBS and counted with trypan blue presence. 2.5 x 10^6^ of live cells were placed in Eppendorf tubes washed in FACS wash buffer and centrifugated (500 x G; 10 min. 20˚C). The washed cells were then blocked in blocking buffer (anti-CD16/CD32 mAb Cat. No 553142; BD Biosciences, Germany) and incubated with the fluorophore conjugated antibodies for 30 min at 4°C. Antibodies and their isotype controls for background staining were ordered from BD Biosciences, with the exception of eBioscience Mouse Regulatory T Cell Staining Kit (Cat. No 88-8118-40), purchased from Invitrogen (USA). To analyse the phenotype of isolated lung cells the following surface markers were examined: macrophages (CD11b/Mac-1, Cat. No 553310; F4/80, Cat. No 565410), lymphocytes Th (CD3, Cat. No 555275; CD4, Cat. No 553650), lymphocytes Tc (CD3, Cat. No 555275; CD8, Cat. No 553035), lymphocytes Treg (CD4, Cat. No 553650; Foxp3, Cat. No 88-8118-40), lymphocytes B (CD19, Cat. No 553785; CD45R/B220, Cat. No 553089), lymphocytes NK (CD161c, Cat. No 550627). First, the cells were gated based on FSC and SSC plot. The events with very low FSC and SSC were eliminated. In the next step, the expression of investigated markers was determined by means of two-parameter density plots. To exclude background and false-positive staining, the gates for individual markers were inserted based on the isotype control signal. Specific isotype controls were used for each marker tested. To determine the amount of Treg cells in lung tissue, after excluding cell debris and dead cells with FSC vs SSC plot, the CD4 cells were gated using a histogram plot. The gate was set based on isotype control for CD4 staining. Then, by means of two-parameter density plot, markers CD4 and FoxP3 were tested. Specific isotype controls were used for each marker tested. The isotype for FoxP3 was added after fixation and permeabilization of the cells. Samples were analyzed using a FACS Calibur flow cytometer (BD Biosciences). The signal was read from a 1x10^6^ live cells. FlowJo software (FlowJo LLC) was used to analyse the expression of surface markers. The FACS gating strategy was presented in S2 Fig.

### Statistical analysis

The data were presented as the mean value and standard error of the mean (SEM). Statistical analysis was performed using linear regression analysis, as well as one way-ANOVA with Tukey *post-hoc* test, and column statistics used for comparisons. Significance was accepted at p < 0.05.

## Results

### CRAMP physiological concentration and its changes in response to inhalation with synthetic cathelicidin

**Fig 1:**
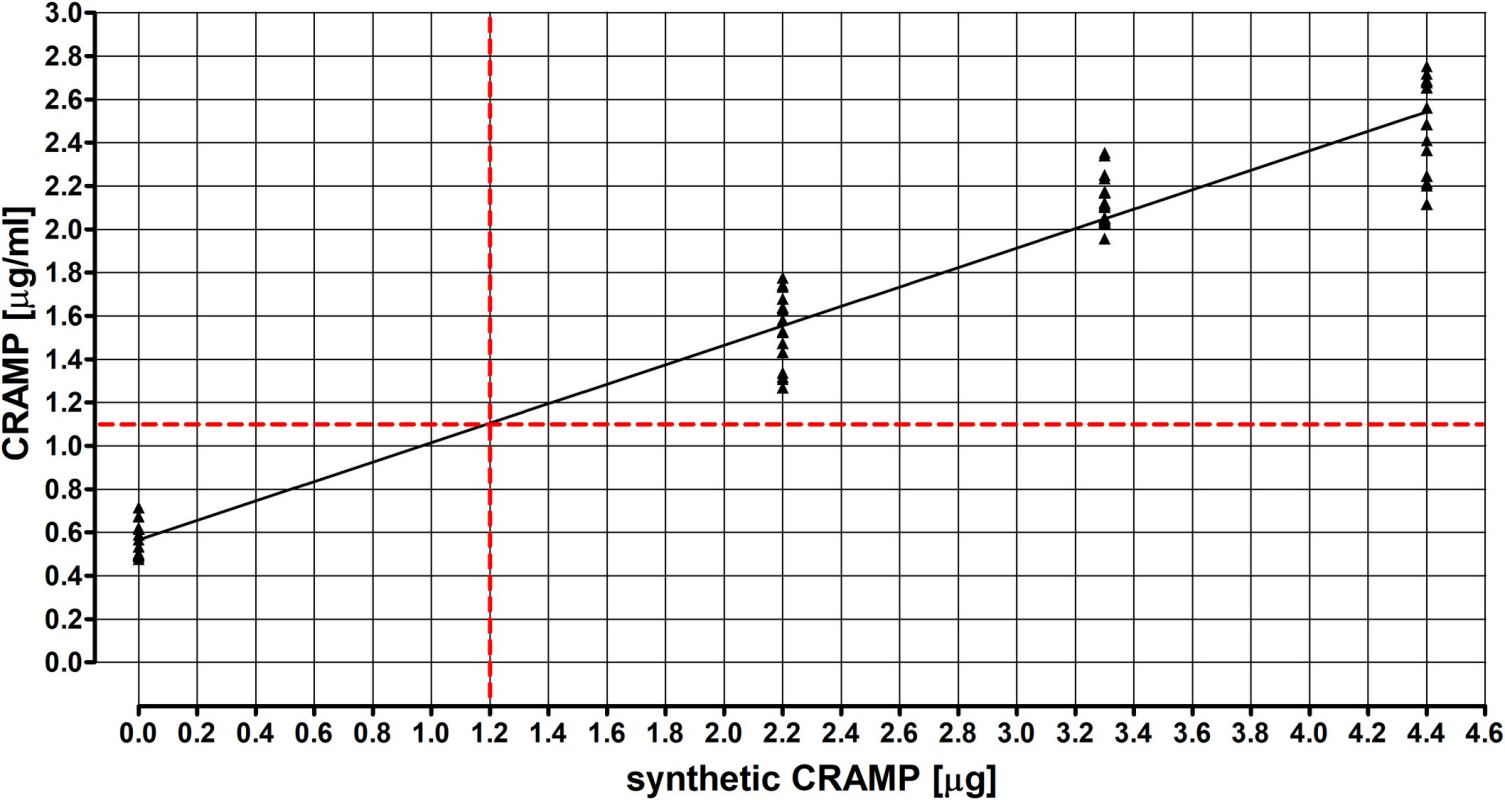
Relationship between lung tissue concentration of CRAMP and dose of synthetic cathelicidin use for inhalation. Protein concentration was investigated in homogenates of mouse lungs collected from untreated mice and from animals after a single exposure to synthetic cathelicidin at concentration 4, 6 or 8 times higher than physiological. Each single inhalation included 1 h exposure to PBS and 1 h exposure to appropriate concentration of synthetic cathelicidin. CRAMP concentration was determined using the ELISA method. Linear regression analysis of CRAMP concentration in response to synthetic cathelicidin inhalation was conducted. Studies were conducted in 4 research groups, each of which contained 4 animals. Each sample was analyzed in 4 replications. (control mice N = 4; treated mice per research group N = 4).

The physiological concentration of CRAMP in lung tissue homogenates was 0.553 μg/ml (Fig 1). Mice single exposure to synthetic cathelicidin at concentration 4, 6 or 8 times higher than physiological increase CRAMP concentration to 1.538, 2.129 and 2.489 μg/ml. Linear regression analysis of CRAMP concentration in response to synthetic cathelicidin inhalation revealed that doubling the physiological concentration of cathelicidin in the lung tissue (1.106 μg/ml) requires the administration to mice in a single inhalation of 1.2 μg of synthetic defence peptide.

### Changes in composition of immune cells in murine model of hypersensitivity pneumonitis in response to cathelicidin treatment

**Fig 2:**
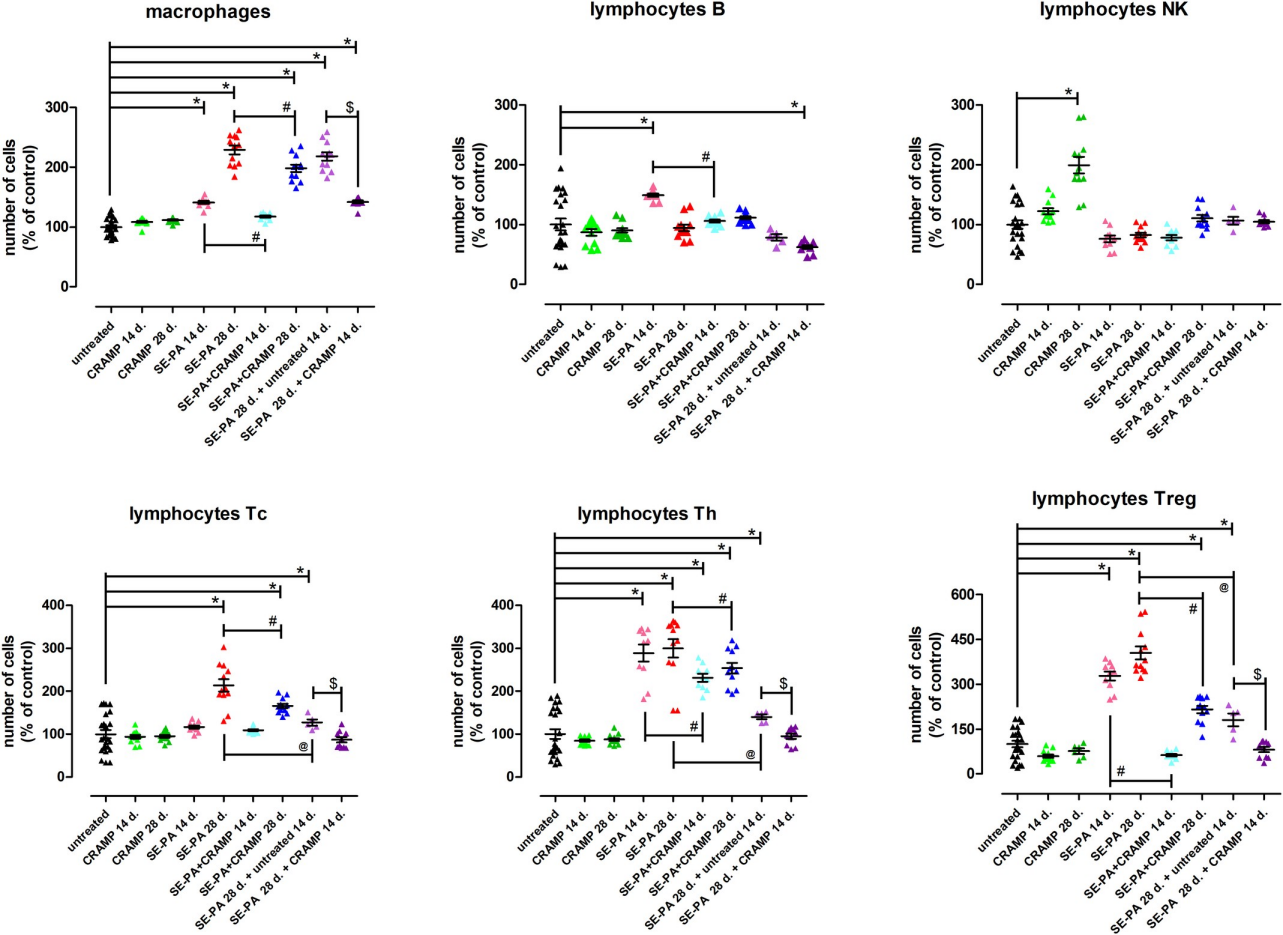
Changes in immune cells composition in response to cathelicidin (CRAMP) and/or Saline Extract of *Pantoea Agglomerans* (SE-PA) treatment. Cells number was investigated in lungs collected from untreated mice and mice exposed to investigated compounds for 14, 28 or 42 days using flow cytometry. The data are presented as mean of cell number ± SEM. Each research group contained 8 mice: 6 treated and 2 untreated animals. Samples were collected from all animals and analyzed in 2 replications. (control mice N = 16; treated mice per research group N = 6). * p < 0.05 vs. untreated; # p < 0.05 SE-PA+CRAMP 14 d./28 d. vs. SE-PA 14 d./28 d. (comparison within corresponding time points); @ SE-PA 28 d. + untreated 14 d. vs. SE-PA 28 d.; $ p < 0.05 SE-PA 28 d. + CRAMP 14 d. vs. SE-PA 28 d. + untreated 14 d.; one-way ANOVA test; Tuckey *post-hoc* test.

In mice, chronic exposure to cathelicidin did not impact on the level of macrophages and lymphocytes: B, Tc, Th and Treg (Fig 2). On the contrary, a significant accumulation of Natural Killer (NK) cells (around 100% vs. control) was observed in lung tissues collected from animals treated with CRAMP for 28 days. However, the NK cells number was not affected by exposure to SE-PA or SE-PA in combination with CRAMP. Significant changes in the level of immune cells (except NK cells) were observed in response to SE-PA treatment, and the range of alterations was dependent on exposure time. The number of macrophages, lymphocytes Treg and lymphocytes Th increased gradually as the exposure period was extended. The following cell numbers were observed at investigated time points (14 days of exposure: 141%, 327%, 289%; 28 days of exposure: 229%, 404%, 300%). The number of other investigated lymphocytes was also affected by SE-PA treatment; however, significant accumulation of lymphocytes B was observed only after 14 days of exposure (149%), while lymphocytes Tc number significantly increased (214%) in response to 28 days of SE-PA treatment. It needs to be highlighted that elevated levels of lymphocytes Tc, lymphocytes Treg and lymphocytes Th observed in lung tissue of mice exposed for 28 days to SE-PA clearly decreased after cessation of antigen exposure, but was still higher than the control level. Simultaneously, the number of macrophages was not affected by the cessation of SE-PA exposure, and in both compared research groups (SE-PA 28 d. + untreated 14 d. vs SE-PA 28 d.) was more than twice as high as in the control. Cathelicidin administered together with SE-PA and after SE-PA treatment effectively decreased the level of macrophages and lymphocytes elevated by inhalation with bacterial antigen. The most spectacular preventive effect was observed in the case of lymphocytes Treg, which amounted after 14 and 28 days of CRAMP administration together with SE-PA decreased, respectively, by 265% and 189%, compared with the levels recorded in animals exposed only to SE-PA. The strongest therapeutic effect was also observed in the case of lymphocytes Treg; the difference in the investigated number of cells between lungs collected from mice exposed for 28 days to SE-PA and then treated for 14 days with CRAMP vs. mice exposed for 28 days to SE-PA and then untreated for 14 days was 100%. A similar therapeutic effect was observed in the case of macrophage, and the recorded difference between the above-mentioned research groups was 76%. Furthermore, the beneficial impact of cathelicidin on the number of macrophages altered by SE-PA inhalations was more evident when CRAMP was given after SE-PA treatment, than together with antigens. It has to be highlighted that cathelicidin treatment was not able to completely neutralize negative changes in the number of immune cells induced by SE-PA; however, in the case of lymphocytes Tc, lymphocytes Treg and lymphocytes Th, CRAMP administration after cessation of SE-PA exposure decrease the number of analyzed cells to the level observed in the untreated mice. The representative dot blots for changes presented in Fig 2 was shown in S3 Fig.

### Cathelicidin inhibited cytokine production in mouse model of hypersensitivity pneumonitis

**Fig 3:**
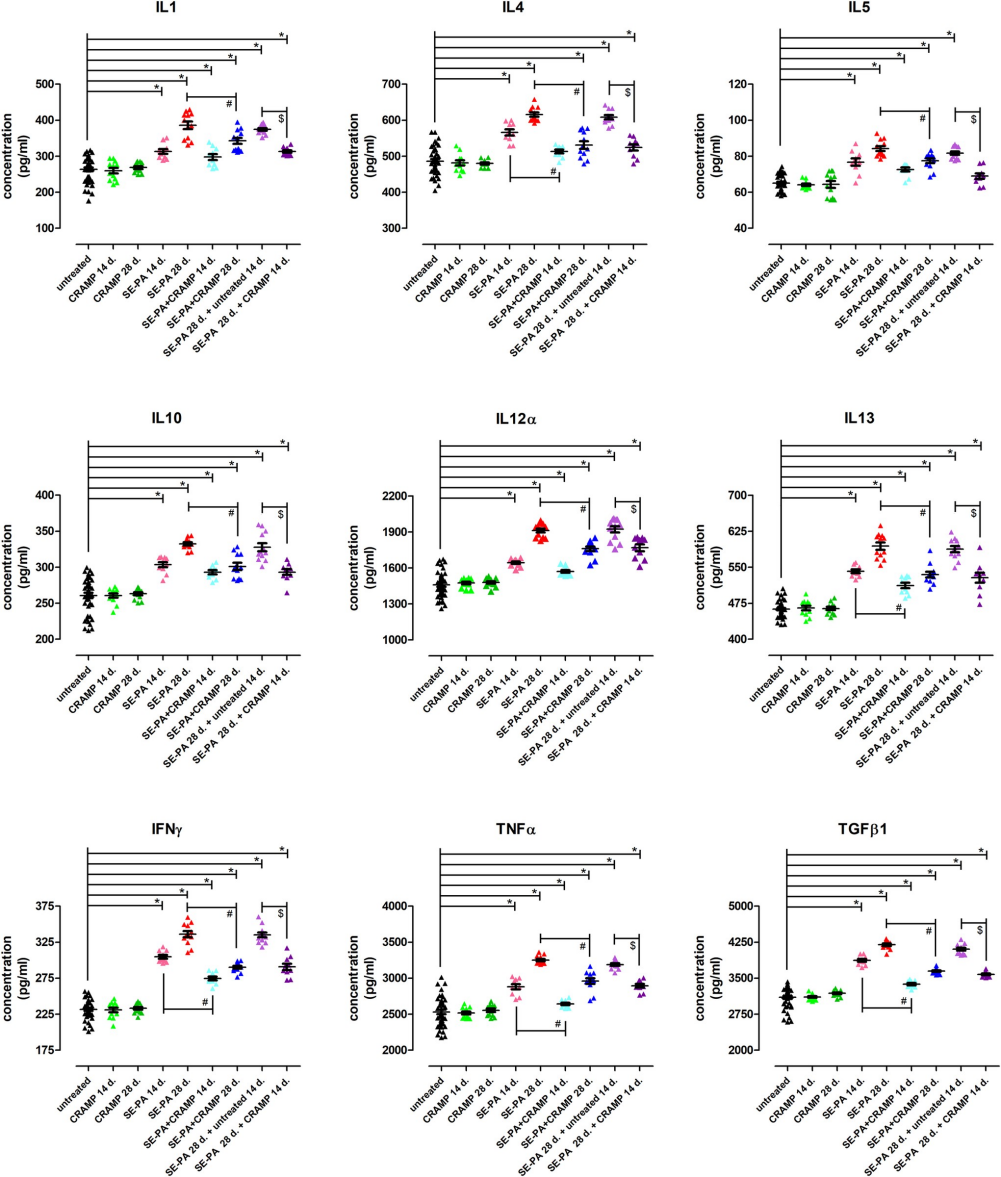
Changes in cytokines expression induced by cathelicidin (CRAMP) and/or Saline Extract of *Pantoea Agglomerans* (SE-PA) treatment. Protein concentration was investigated in homogenates of lungs collected from untreated mice and animals exposed to investigated compounds for 14, 28 or 42 days using the ELISA method. The data are presented as a mean of protein concentration ± SEM. Each research group contained 8 mice: 6 treated and 2 untreated animals. Samples were collected from all animals and analyzed in 2 replications. (control mice N = 20; treated mice per research group N = 6). * p < 0.05 vs. untreated; # p < 0.05 SE-PA+CRAMP 14 d./28 d. vs. SE-PA 14 d./28 d. (comparison within corresponding time points); @ SE-PA 28 d. + untreated 14 d. vs. SE-PA 28 d.; $ p < 0.05 SE-PA 28 d. + CRAMP 14 d. vs. SE-PA 28 d. + untreated 14 d.; one-way ANOVA test; Tuckey *post-hoc* test.

Chronic exposure of mice to cathelicidin did not change, compared to the control, concentrations of the following cytokines: IL1β, IL4, IL5, IL10, IL12α, IL13, IFNγ, TNFα, TGFβ1 (Fig 3). Up-regulation of expression of the mentioned proteins was noted in mouse lungs in response to SE-PA treatment, and the observed effect was dependent on exposure time. The most significant changes were observed in the case of IL1β and IFNγ, the expression of which increased after 14 and 28 days of exposure to bacterial extract, on average, 25% and 46%, compared to untreated mice. The increase of expression of other investigated cytokines in response to SE-PA was 116% of control and 129% of control in the analysed time points. The cessation of mice exposure to *P*. *agglomerans* extract did not cause statistically significant changes in the expression of examined cytokines, in comparison to their expression recorded in mice treated with SE-PA for 28 days. Analysis of cytokines profiles revealed that cathelicidin administered after SE-PA exposure decreased the level of all investigated cytokines elevated by the bacterial extract, but was not able to restore them to their normal level. The average difference in the level of protein expression in the analysed groups was 16%, the strongest therapeutic effect of CRAMP was observed in the case of IL1β, the expression of which was reduced by 23%. A beneficial effect of cathelicidin was also observed when defence peptide was provided together with SE-PA; however, 2 patterns of CRAMP response were observed: 1) IL4, IL13, IFNγ, TNFα, TGFβ1: statistically significant differences in the level of cytokines expression after 14 and 28 days of animals exposure to SE-PA used alone and together with CRAMP (average differences were 11% at time point 7 days, and 16% at time point 14 days); 2) IL1β, IL5, IL10, IL12α: statistically significant differences in the level of cytokines expression after 28 days of animals exposure to SE-PA used alone and together with CRAMP (average difference 17%). It has to be highlighted that cathelicidin administered together with SE-PA was not able to completely eliminate the antigen-induced changes in the level of analyzed cytokines.

### Cathelicidin reduced inflammation and fibrosis in murine model of hypersensitivity pneumonitis

**Fig 4:**
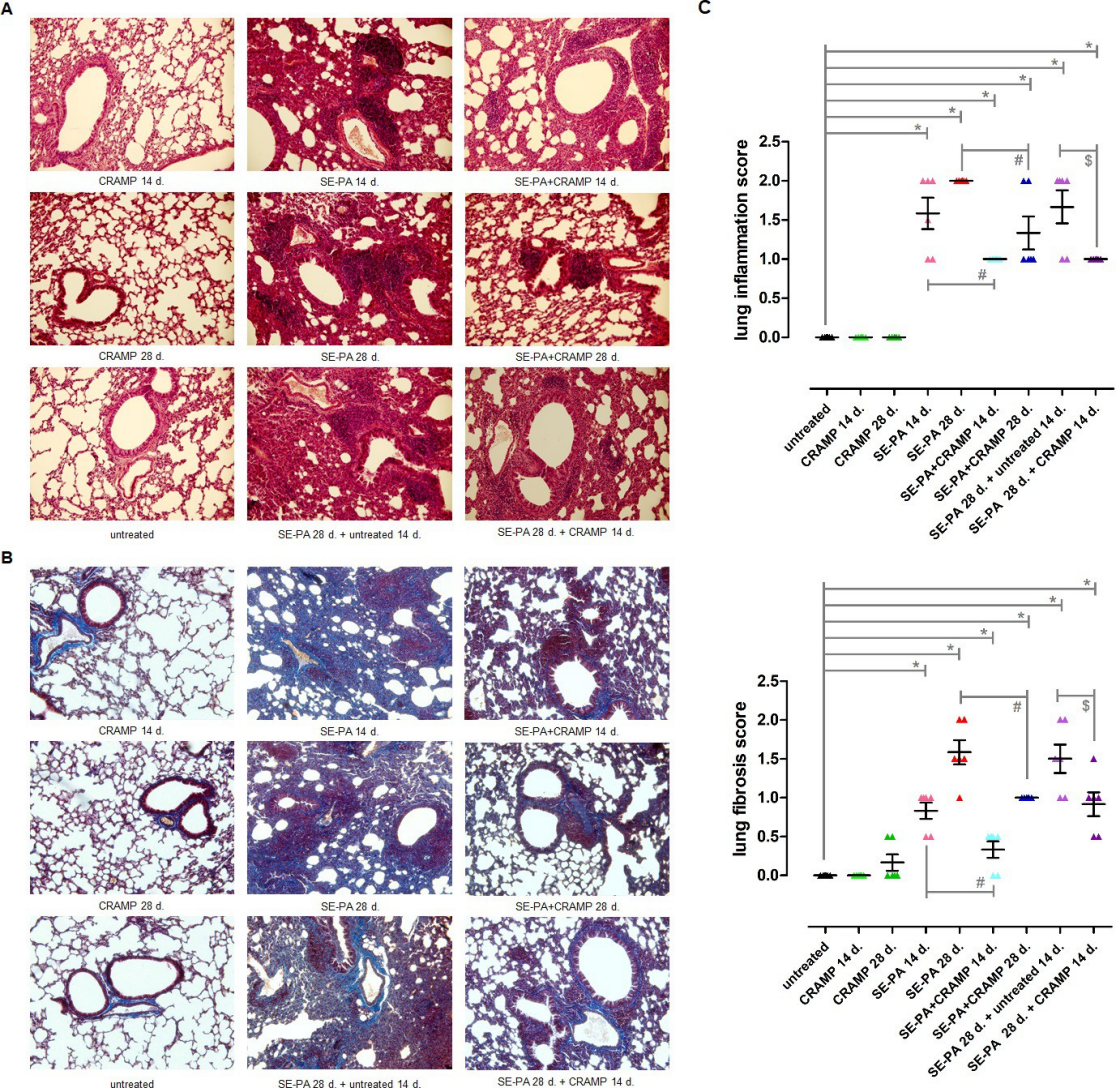
Changes in the morphology of lung tissue of mice exposed to cathelicidin (CRAMP) and/or Saline Extract of *Pantoea Agglomerans* (SE-PA). Lungs collected from untreated mice and animals exposed to investigated compounds for 14, 28 or 42 days were stained with haematoxylin and eosin (H&E), as well as Masson trichrome dyes (TRI), and examined under light microscopy at magnification 200×. Each research group contained 8 mice: 6 treated and 2 untreated animals. Samples were collected from all animals and analyzed. (control mice N = 16; treated mice per research group N = 6). A) Representative photographs of mouse lung sections stained with H&E. B) Representative photographs of mouse lung sections stained with TRI. C) Quantification of inflammation and fibrosis in collected mouse lung tissue after H&E and TRI staining. The histological scores were graded with 4 point scales: 0 = regular tissue, 1 = mild changes, 2 = moderate changes, 3 = significant changes. The data for histologic scores for 2 independent investigations are given as mean ± SEM of investigated items. * p < 0.05 vs. untreated; # p < 0.05 SE-PA+CRAMP 14 d./28 d. vs. SE-PA 14 d./28 d. (comparison within corresponding time points); $ p < 0.05 SE-PA 28 d. + CRAMP 14 d. vs. SE-PA 28 d. + untreated 14 d.; one-way ANOVA test; Tuckey *post-hoc* test.

Histological examination of mouse lung sections stained with H&E and Masson trichrome dyes revealed that mice exposed to cathelicidin for both 14 days and 28 days did not cause any changes in the lung tissue morphology (Fig 4). On the contrary, centrolobe and interstitial inflammation with marked infiltration of lymphocytes, as well as alveolar distortion and thickening of alveolar walls, were observed after 14 days of SE-PA exposure. Quantification of lung injury in the research group showed the following mean scores for inflammation and fibrosis: 1.6 and 0.8, respectively. Additional exposure of mice to SE-PA increased the inflammatory response (mean score for inflammation: 2.0). Furthermore, the respiratory ducts showed significant obstruction of the lumen due to lymphocyte and macrophage infiltration, as well as abnormal collagen deposition (mean score for fibrosis 1.6). The lungs collected 14 days after cessation of mice chronic (28 days) exposure to SE-PA also manifested inflammatory reaction (mean score for inflammation: 1.7) and signs of fibrosis (mean score for fibrosis: 1.5). It should be noted that the decrease in the inflammation severity observed in the mentioned research group in comparison to results obtained from lungs collected from mice just after 28 days of exposure to SE-PA, was not statistically significant. On the other hand, CRAMP administered together with bacterial extract silenced inflammatory responses, which was expressed as a reduction in inflammation scores by 0.6 (SE-PA+CRAMP 14 d.) and 0.7 (SE-PA+CRAMP 28 d.), compared to mice which inhaled SE-PA for 14 days and 28 days, respectively. Nevertheless, infiltration of immune cells was still evident. Furthermore, cathelicidin administration together with SE-PA inhibited the induction of fibrosis by 0.5 (SE-PA+CRAMP 14 d.) and 0.6 (SE-PA+CRAMP 28 d.), compared to results observed in mice that inhaled only the bacterial extract for 2 and 4 weeks, respectively. Histological examination of the lungs collected from mice treated 14 days with cathelicidin after cassation of SE-PA chronic exposure, revealed lymphocyte infiltration (mean score for inflammation: 1.0), as well as abnormal collagen accumulation (mean score for fibrosis: 0.9). Comparison of the above-mentioned results with changes observed in mouse lungs 14 days after cessation of antigen exposition, showed a statistically significant reduction in inflammatory response and degree of fibrosis.

### Cathelicidin inhibited collagen deposition in murine model of hypersensitivity pneumonitis

**Fig 5:**
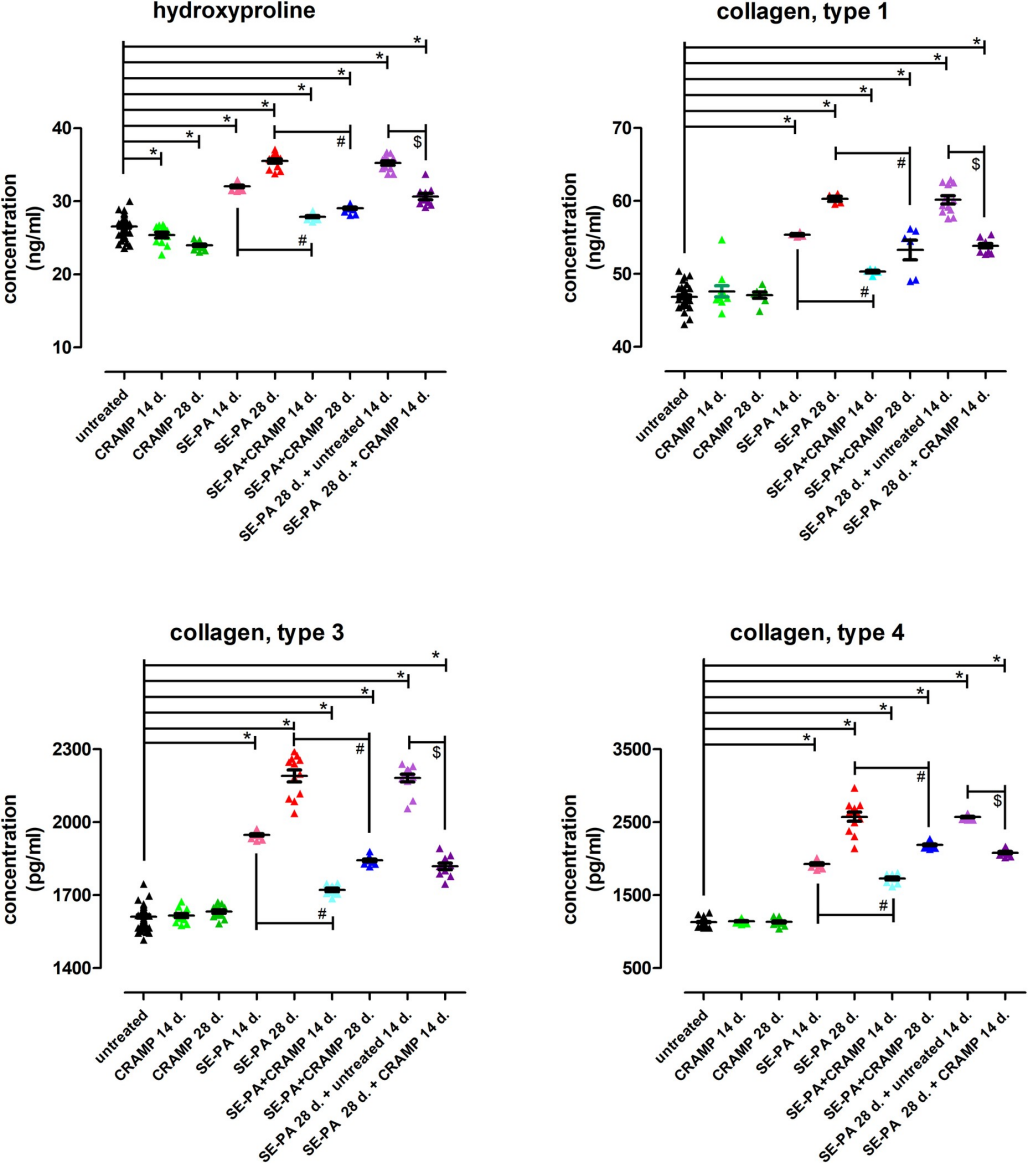
Alterations in collagen deposition in response to cathelicidin (CRAMP) and/or Saline Extract of *Pantoea Agglomerans* (SE-PA) treatment. Protein concentration was investigated in homogenates of lungs collected from untreated mice and animals exposed to investigated compounds for 14, 28 or 42 days using the ELISA method. The data are presented as a mean of protein concentration ± SEM. Each research group contained 8 mice: 6 treated and 2 untreated animals. Samples were collected from all animals and analyzed in 2 replications. (control mice N = 20; treated mice per research group N = 6). * p < 0.05 vs. untreated; # p < 0.05 SE-PA+CRAMP 14 d./28 d. vs. SE-PA 14 d./28 d. (comparison within corresponding time points); @ SE-PA 28 d. + untreated 14 d. vs. SE-PA 28 d.; $ p < 0.05 SE-PA 28 d. + CRAMP 14 d. vs. SE-PA 28 d. + untreated 14 d.; one-way ANOVA test; Tuckey *post-hoc* test.

Cathelicidin used alone did not cause any changes in the collagens concentration, on the contrary, a significant decrease in hydroxyproline concentration was observed (Fig 5). Chronic exposure of mice to SE-PA revealed time-dependent collagen, as well as hydroxyproline accumulation in lung tissues. The most significant changes were noted in the case of collagen type 4, in which the concentration increased during SE-PA inhalations to 170% and 227% of control, respectively. Nevertheless, taking into account absolute values, the largest alterations were observed in the level of collagen type 1, in which the concentration increased by 8.5 ng/ml and 13.4 ng/ml after 14 and 28 days of SE-PA treatment. It must be noted that the concentration of investigated proteins increased by SE-PA remained at a comparable, high level even after the cessation of animals exposure to antigen. Cathelicidin administered together with SE-PA or after SE-PA treatment evidently decreased the elevated level of collagens and hydroxyproline. Nevertheless, CRAMP was not able to completely inhibit collagen and hydroxyproline deposition in the lungs of mice exposed to SE-PA. After 14 and 28 days of defence peptide administration together with *P*. *agglomerans*, the concentration of hydroxyproline and collagens, was reduced, on average, by 11% and 15%, compared to the levels of investigated compounds observed in mice treated only with SE-PA. Similarly, 14 days of CRAMP administration after 28 days of mice exposure to SE-PA, decreased the level of collagens and hydroxyproline, on average, by 15%, compared to proteins concentration noted in the lungs of mice which inhaled *P*. *agllomerans* extract for 28 days.

### Cathelicidin impact on mouse pulmonary function in physiological and pathological conditions

**Fig 6:**
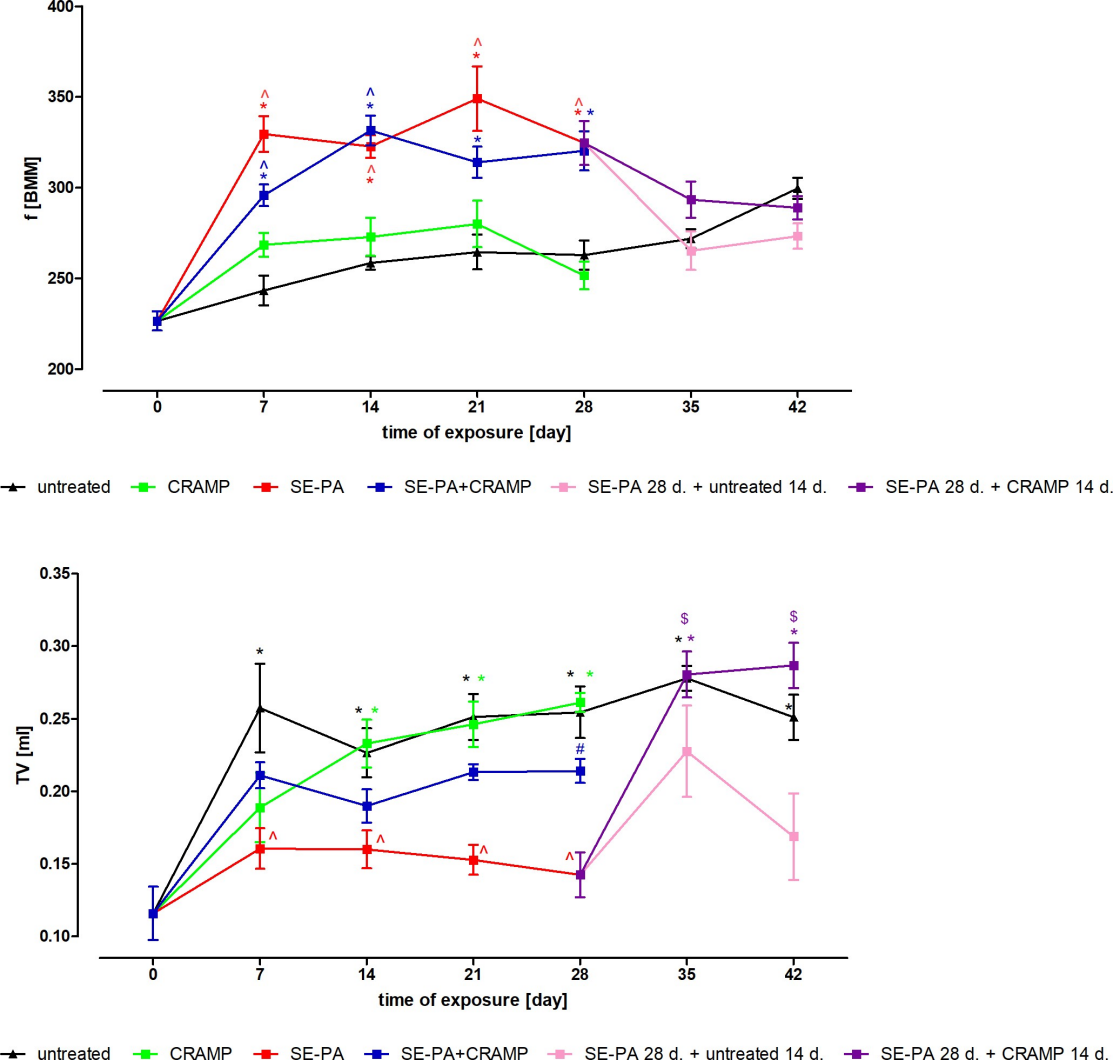
Changes in mouse pulmonary function in response to cathelicidin (CRAMP) and/or Saline Extract of *Pantoea Agglomerans* (SE-PA) treatment. The frequency of breathing (f) and tidal volume (TV) was investigated before treatment and after every 7 days of mice exposure to investigated compounds. The data were collected every 2 s for 15 min. The data are presented as a mean of tested ventilatory parameters ± SEM. Each research group contained 8 mice: 6 treated and 2 untreated animals. A pulmonary function test was conducted in all animals. * p < 0.05 vs. untreated; ^ treated vs. untreated (comparison within corresponding time points); # p < 0.05 SE-PA+CRAMP vs. SE-PA (comparison within corresponding time points); @ p < 0.05 SE-PA 28 d. + CRAMP 14 d. vs. SE-PA 28 d. + untreated 14 d. (comparison within corresponding time points); $ p < 0.05 SE-PA 28 d. + CRAMP 14 d./ SE-PA 28 d. + untreated 14 d vs. SE-PA (day 28). One-way ANOVA test; Tuckey *post-hoc* test.

The frequency of breathing of the untreated mice was similar to that of mice exposed to cathelicidin (Fig 6). On the contrary, SE-PA treatment increased the respiratory rate in the mice tested by around 30%, compared to non-inhaled animals, and more than 20% compared to animals treated with CRAMP. Cathelicidin administration together with SE-PA did not cause statistically significant changes in respiratory frequency, compared to the group exposed to *P*. *agglomerans* extract. Cessation of animals’ inhalation with SE-PA, as well as administration of CRAMP after 4 weeks of exposure to the *P*. *agglomerans* extract, resulted in a reduction in respiratory rate to the level observed in untreated mice.

The largest changes in the tidal volume were observed between day 0 and day 7 of the experiment in untreated animals—TV value increased from 0.116 ml to 0.257 ml. Between days 7 and 28 of the experiment, no statistically significant differences in TV values were observed in mice which inhaled CRAMP alone, CRAMP with SE-PA, or the control group. A comparison of the TV values between untreated mice and animals exposed to SE-PA showed a TV reduction of more than 30% in response to the test extract. The combined administration of SE-PA and CRAMP resulted in an increase in TV value compared to the mice exposed to SE-PA, but statistically significant differences were observed only after 28 days of inhalation. A beneficial impact of cathelicidin on TV was also observed when the defence peptide treatment was implemented after 4 weeks of mice inhalation with SE-PA. TV recorded in the mentioned research group (SE-PA 28d. + CRAMP 14 d.) was similar to changes noted in the group of non-inhaled animals.

## Discussion

Several cathelicidin features, e.g. antimicrobial [18–20], immunoenhancement [17, 18], proinflammatory [17, 21–25, 37] and wound healing promotion [26–28], make this host defence peptide an ideal candidate for use in the treatment of hypersensitivity pneumonitis. On the other hand, due to the importance of cathelicidin for the mechanisms of innate immunity, every therapeutic strategy which can interfere with the LL-37 level may lead to worsening of the patient’s condition. Furthermore, cathelicidin has only a moderate selectivity for bacterial cells or other pathogen cells, and may cause permeabilization of host cells membrane inducing their necrosis or apoptosis at concentrations not much higher than antipathogenic ones. The cytotoxic effects of cathelicidin were observed in different cell types, including lymphocytes, erythrocytes, epithelial cells and fibroblasts [28, 38–41]. Consequently, the selection of the cathelicidin concentration suitable for the current study was a challenge. The basis for the decision were the results of the authors previous clinical and *in vivo* studies. Examination of changes of cathelicidin concentration in the course of respiratory pathologies and due to exposure to organic dust indicated a decrease of the cathelicidin levels in the human pulmonary compartment in cases of pulmonary fibrosis [29, 42] and advanced stages of COPD [32]. Additional studies conducted in the HP model revealed both significant down-regulation of CRAMP gene expression (Fold Change = - 2.28) and a decrease of cathelicidin concentration (Fold Change = - 1.66) in mouse lungs collected after 28 days of exposure to *P*. *agglomerans*, compared to untreated mice [20]. Based on the cited results, it was established that the optimal will be cathelicidin application in a concentration needed to obtain in mice lung tissue a twice as high concentration than the physiological. Conducted dose-response studies have demonstrated the need to give CRAMP in a dose of 1.2 μg/mouse/inhalation to achieve the intended effect. According to expectation, this concentration should only restore the balance impaired by exposure to organic dust without toxic lesions in the respiratory tract.

In the first step of the study, changes in immune cell composition in collected lung tissues were analysed. Flow cytometry revealed that chronic exposure of mice to cathelicidin did not affect the number of macrophages, lymphocytes T (Th, Tc, Treg) or lymphocytes B, but at the same time in lungs collected after 28 days of inhalation with CRAMP, a doubling in the number of NK cells was observed. The significant accumulation of NK cells in response to chronic cathelicidin treatment may be explained by the enhancement of NK cells proliferation by the investigated peptide, which has been proven in several studies [43, 44]. Nevertheless the lack of statistically significant changes in the number of NK cells after 14 days of mice exposure to CRAMP weakens this hypothesis. The doubling in the number of NK cells observed in mice after 4 weeks of inhalation with synthetic peptide may have been caused by the NK cell-specific ability to recognize foreign proteins (synthetic CRAMP), without prior immunization, which is followed by NK cells activation associated with their influx and increased proliferation at the location of the foreign protein [45]. A weak point of this hypothesis, similar to the previously presented explanation, is also the time when this reaction was observed, as well as the fact of use in the research synthetic cathelicidin of a consensus sequence of mouse CRAMP. In the authors’ opinion, the lack of changes in the investigated immune cell number (except NK cells) in response to cathelicidin treatment indicates the proper selection of synthetic peptide dose used in the study. Even NK cells accumulation induced by CRAMP chronic exposure should not be a concern because of the specificity and selectivity of NK cells action [46]. Furthermore, because of the possible antifibrotic role of NK cells [47, 48], the significant increase of their number in response to chronic CRAMP exposure could be the basis of beneficial effects of cathelicidin described below. With the except of the mentioned alterations, the number of NK cells was not affected by exposure to SE-PA or SE-PA in combination with CRAMP. On the contrary, mice chronic exposure to SE-PA distinctly increased the number of macrophages and lymphocytes, which was also observed during microscopic observation of lung sections. Immune cell accumulation in response to SE-PA proceeded in two different ways. Alterations in the number of B cells were observed only after 14 days of mice treatment with *P*. *agglomerans*, while the number of Tc, Treg, Th and macrophages increased in a time-dependent manner and was particularly high in the case of Th (3-fold increase) and Treg (4-fold increase). The recorded accumulation of lymphocytes and macrophages and the observed pattern of changes correspond with clinical pictures of HP, wherein the acute and subacute phases are dominated by Type III hypersensitivity reactions typical for (immune complexes mediated), while the chronic phase is characterized by Type IV hypersensitivity reactions (cell-mediated) [1]. Furthermore, the recorded changes are in line with the authors’ previous description of the mouse HP model [14, 15]. It should be noted that changes in the number of immune cells induced by 28 days of mice exposure to SE-PA after cessation of exposure for 2 weeks remained at a similar level only in the case of macrophages, while the number of other investigated lymphocytes (Tc, Treg, Th) decreased evidently but still deviated compared to untreated mice. Cathelicidin administered together and after SE-PA treatment reduced the level of immune cells elevated by harmful extract treatment. Inhibition of lymphocytes accumulation was more effective when cathelicidin was given together with SE-PA, while in the case of macrophages, the biggest reduction in cell numbers was observed when defence peptide was applied after 28 days of SE-PA treatment. Despite the above-mentioned fact, that just cessation of mice exposure to *P*. *agglomerans* decreases the number of Tc, Treg and Th it must be noted that cathelicidin administration after SE-PA exposure restored the immune balance by reducing the elevated level of mentioned lymphocytes.

In the next step, the concentration of cytokines involved in inflammatory and fibrosis process (IFNγ, TNFα, TGFβ1, IL1β, IL4, IL5, IL10, IL12α, IL13) [49] was examined in obtained mouse lungs using the ELISA method. Despite the fact that the impact of cathelicidins on various cell types, such as macrophages, lymphocytes and epithelial cells, inducing the synthesis of different cytokines [17, 50], the profile of the investigated cytokines in the homogenates of lungs collected from mice after 14 days and 28 days of CRAMP treatment was similar to that observed in untreated animals. Once again, the authors have proved that the cathelicidin concentration indicated for the presented study was both adequate and safe. Furthermore, time-dependent accumulation of all tested cytokines (both proinflammatory and profibrotic) in response to SE-PA exposure corresponded with the authors previous data showing the coexistence of inflammation and progressive pulmonary fibrosis [14]. Additionally, cytokines concentration detected after 28 days of *P*. *agglomerans* treatment was similar to data obtained 14 days after completion of the mentioned exposure. Similar levels discovered of monokines (TNFα, TGFβ1, IL1β, IL10, IL12α) in the following research groups: SE-PA 28 d. *vs* SE-PA 28 d. + untreated 14 d., correspond with macrophages accumulation observed in both flow cytometry and microscopic observation [51]. On the other hand unchanged concentration of tested lymphokines (IFNγ, IL4, IL5, IL13), despite the significant decrease of Th cell number [60] in response to the cessation of mice chronic exposure to SE-PA may suggest enhancement of lymphocyte activation leading to increase of tested cytokine release. It should also be noted that the induced by *P*. *agglomerans* significant increase of concentration of antifibrotic cytokines, such as IL10 [52], IL12 [53] and IFNγ [54] does not match the pulmonary fibrosis observed in the histological analysis. However, this atypical cytokine profile has already been described for the murine HP model used in the study [14]. All alterations in cytokines release observed in mice which inhaled SE-PA were largely neutralized by CRAMP administration. Nevertheless, the defence peptide was not able to completely reverse the pathological changes. The beneficial impact of cathelicidin was noted in all animals given the peptide together with *P*. *agglomerans* for 28 days and in all animals that received the peptide after cessation of SE-PA exposure. CRAMP administered together with SE-PA for 14 days also decreased the level of tested cytokines (SE-PA+CRAMP 14 d. vs SE-PA 14 d.) accelerated by *P*. *agglomerans*, but a statistical significance was reported only in the case of IL4, IL13, IFNγ, TNFα and TGFβ1. From an HP therapeutic point of view, the most important discovery was the ability of CRAMP to reduce the level of profibrotic cytokines: TGFβ1, TNFα, IL1β, IL4, IL5 and IL13 elevated by SE-PA treatment. First of all, the mentioned cytokines are involved in the manifestation of myofibroblast, which is a key event in pathological tissue repair [55, 56]. There are several sources of myofibroblasts in fibrotic tissues, controlled by a different set of listed cytokines e.g. TGFβ1, IL4, IL5 and IL13, which can drive the differentiation of resident fibroblast and recruited fibrocytes to the myofibroblast in lung tissues [57–61]. Furthermore, TGFβ1 induced epithelial to mesenchymal transition (EMT) leading to epithelial cells differentiation to cells with myofibroblast-like features [62, 63]. Additionally, TGFβ1 driven EMT intensified in the presence of TNFα and IL1β [64–66]. Some studies have also shown that over-expression of either TNFα or IL-1β in mouse lungs leads to spontaneous pulmonary fibrosis [67, 68]. The importance of IL5 for the development of fibrosis is also connected with recruitment, differentiation and activation of eosinophils in order to release of profibrotic cytokines such as TGFβ1 and IL13 [69].

Histological examination revealed that chronic exposure of mice to SE-PA gradually leads to the development of lung fibrosis, accompanied by lung architectural distortion and a substantial increase in collagen deposition. Pathologic lesions induced by SE-PA remained unchanged even after 14 days of cessation of causative extract. Obtained results correspond with earlier studies by the authors of the current study, and are characteristic of the murine model of HP created by the authors [14, 15]. On the other hand, cathelicidin long-term treatment did not cause any changes in the lung tissue morphology or structure, and in particular, had no effect on collagen composition. Nevertheless, a significant decrease in hydroxyproline concentration was observed in homogenates of the investigated lungs which heralded the antifibrotic action of CRAMP. In accordance with expectations, other studies have shown that cathelicidin administration together with SE-PA or after cessation of SE-PA chronic exposure inhibited the development of fibrosis, which was expressed as reduction in the concentration of both collagen and hydroxyproline. Nevertheless, the investigated peptide was not able to completely neutralize negative changes induced by SE-PA; consequently, alveolar distortion and thickening of the alveolar walls in response to abnormal extracellular matrix deposition were still observed. The discovered mild antifibrotic properties of cathelicidin correspond with data obtained by Park et al. and Yoo et al. [70, 71]. Park et al. revealed substantial inhibition of collagen synthesis in human dermal fibroblasts after LL-37 treatment, which suggests a potential role of cathelicidin in fibrosis reduction, especially during the formation of keloids [71]. At the same time, Yoo et al. showed that cathelicidin inhibits colitis-associated colonic fibrosis in chronic TNBS (trinitrobenzene sulphonic acid) and *Salmonella typhimurium* infection murine models. In both mentioned models, the cathelicidin gene (Camp)-expressing lentivirus was administered intravenously; additionally, CRAMP as a peptide was provided in the TNBS model. Furthermore, Yoo et al. reported that LL-37 treatment significantly down-regulated collagen expression in both human colonic fibroblasts and human primary intestinal fibroblasts isolated from patients with Crohn’s disease [70]. Unfortunately, there is no evidence showing the potential role of cathelicidin in the development and progress of pulmonary fibrosis.

Although pulmonary function tests are important tools for describing the phenotypic characteristics of respiratory disease in humans, research conducted on small animals has a number of limitations and restrictions. The most commonly used pulmonary function parameters of humans cannot be investigated in mice, especially in the case of non-invasive methods, which makes it difficult for any analysis of changes observed in animals with a clinical picture of the disease. In the presented study, 2 basic breathing parameters were analysed: frequency of breathing (f) and tidal volume (TV). The performed study revealed a similar pattern of changes in respiratory parameters in mice chronically exposed to cathelicidin and in untreated animals. At the same time, a significant deterioration of tidal volume and frequency of breathing was observed in animals chronically exposed to SE-PA. Despite the fact that cathelicidin administration, together with SE-PA or after 4 weeks of mice exposure to SE-PA, improved the analysed respiratory parameters by bringing their values closer to those observed in the control, a statistically significant improvement was only observed in the case of TV. A beneficial impact of cathelicidin on TV was observed when defence peptide treatment was implemented after 4 weeks of mice inhalation with SE-PA, and also after 28 days of mice exposure to both CRAMP and SE-PA. The weaker and less spectacular than it would appear from the histological analysis, the respiratory response to cathelicidin treatment of *P*. *agglomerans* induced lesions can perhaps explain the specific structure and properties of the mouse respiratory system. Irvin and Bates indicated several anatomic features of mouse lungs which can be significant for lung function e.g. the parenchyma occupies a greater fraction of total mouse lung than that of the human; the thickness of the blood-gas barrier in the mouse is smaller than that of the human, which significantly impacts on gas exchange and the parenchymal lung mechanism; the large airway lumen in the mouse, compared to the human, thus better reduces the flow-resistive load; and mouse lungs are characterized by the absence of submucosal glands [72]. Furthermore, based on previous research results, Irvin and Bates noted that functioning of the respiratory system of healthy animals and animals exposed to antigens differed slightly; consequently, inflammatory processes that could compromise lung function in humans have little effect in mice [72–74]. The current results correspond with the observations of Irvin and Bates, indicating the small compatibility of the results of lung function analyses with the changes demonstrated in the histological and immunological investigations.

## Conclusions

The obtained results indicate that cathelicidin silenced immune responses and inhibited fibrosis induction in the murine model of HP. A beneficial effect of cathelicidin was associated with restoration of the balance in the number of immune cells and cytokines production. Nevertheless, cathelicidin was not able to completely reverse pathological changes. It needs to be highlighted that in the face of the lack of effective and safe methods of preventing and treating pulmonary fibrosis in the course of HP, even a small beneficial effect of cathelicidin, especially in the absence of negative effects, deserves attention and presentation to a wider audience in order to create the basis for developing an effective therapeutic strategy in the future.

## Supporting information

S1 Fig Design of experiments. (TIF)

S2 Fig FACS gating strategy. (TIF)

S3 Fig The representative dot blots for changes presented in Fig 2. (TIF)
