# Supplementary figures and images for: Beneficial impact of cathelicidin on hypersensitivity pneumonitis treatment—In vivo studies
Source: PLoS One. 2021 May 17;16(5):e0251237. doi: 10.1371/journal.pone.0251237 (PMC8128276; doi:10.1371/journal.pone.0251237)

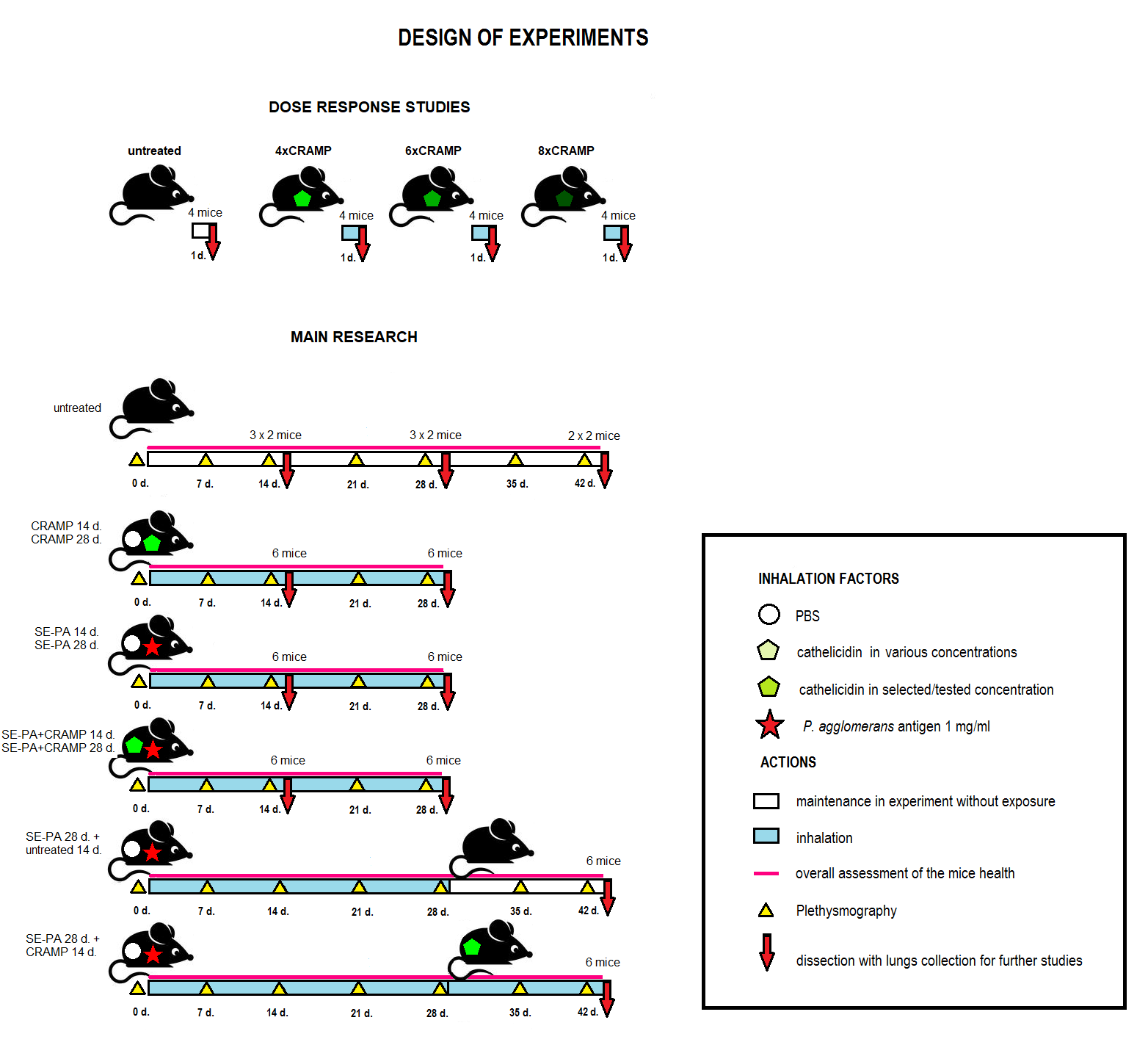

Supplement: S1 Fig — (TIF) [file pone.0251237.s001.tif]

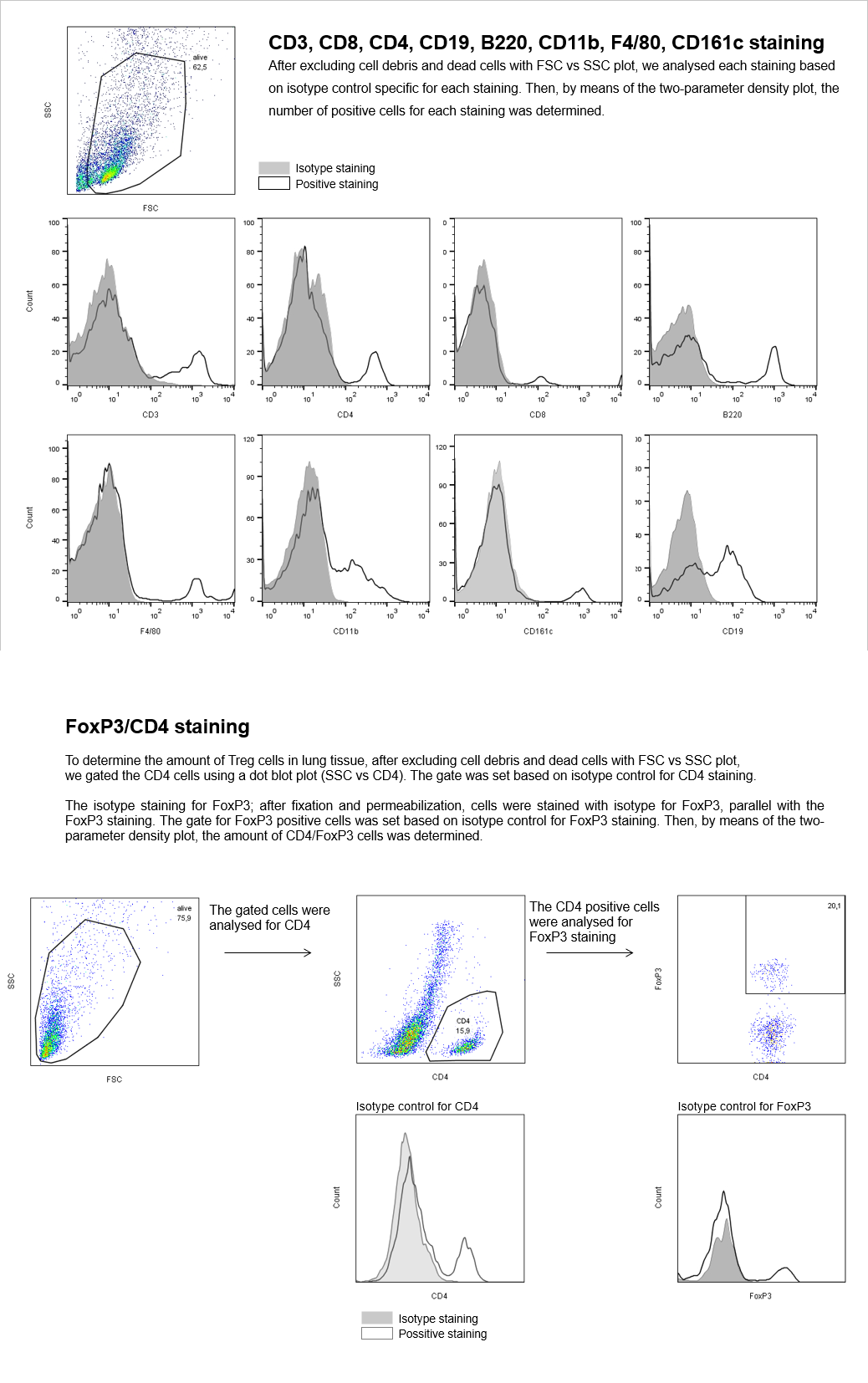

Supplement: S2 Fig — (TIF) [file pone.0251237.s002.tif]

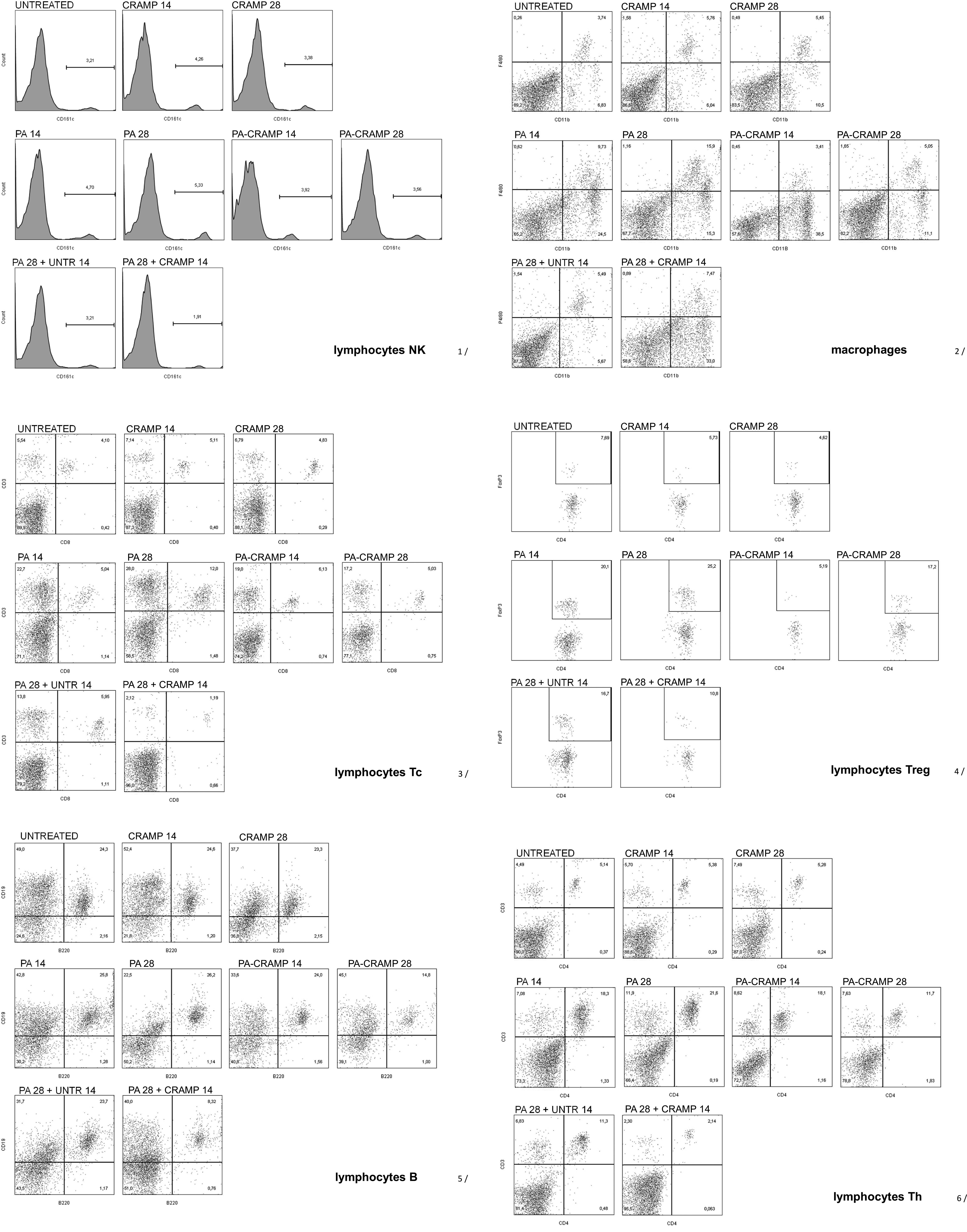

Supplement: S3 Fig — (TIF) [file pone.0251237.s003.tif]
